# Supplementary material for: Clinical progression, disease severity, and mortality among adults hospitalized with COVID-19 caused by the Omicron and Delta SARS-CoV-2 variants: A population-based, matched cohort study
Source: PLoS One. 2023 Apr 27;18(4):e0282806. doi: 10.1371/journal.pone.0282806 (PMC10138229; doi:10.1371/journal.pone.0282806)
Supplement: S1 File — (DOCX) [file pone.0282806.s001.docx]

**Supplementary Figures, Tables and Appendix 1**

**Clinical progression, disease severity, and mortality among patients hospitalized with COVID-19 caused by the Omicron and Delta SARS-CoV-2 variants: A multicenter, matched cohort study**

**Supplementary Figure 1:** STROBE flow diagram for the identification of eligible participants in the study. Risk factors for severe COVID-19 were defined as any of the following conditions: diabetes, body mass index (BMI) >30, chronic heart disease including hypertension, chronic pulmonary disease, chronic kidney disease, chronic liver disease, active cancer, immunosuppression, cerebrovascular disease and pregnancy. Risk factors were counted as number of risk factors in a score of 0, 1, 2 or, 3+.


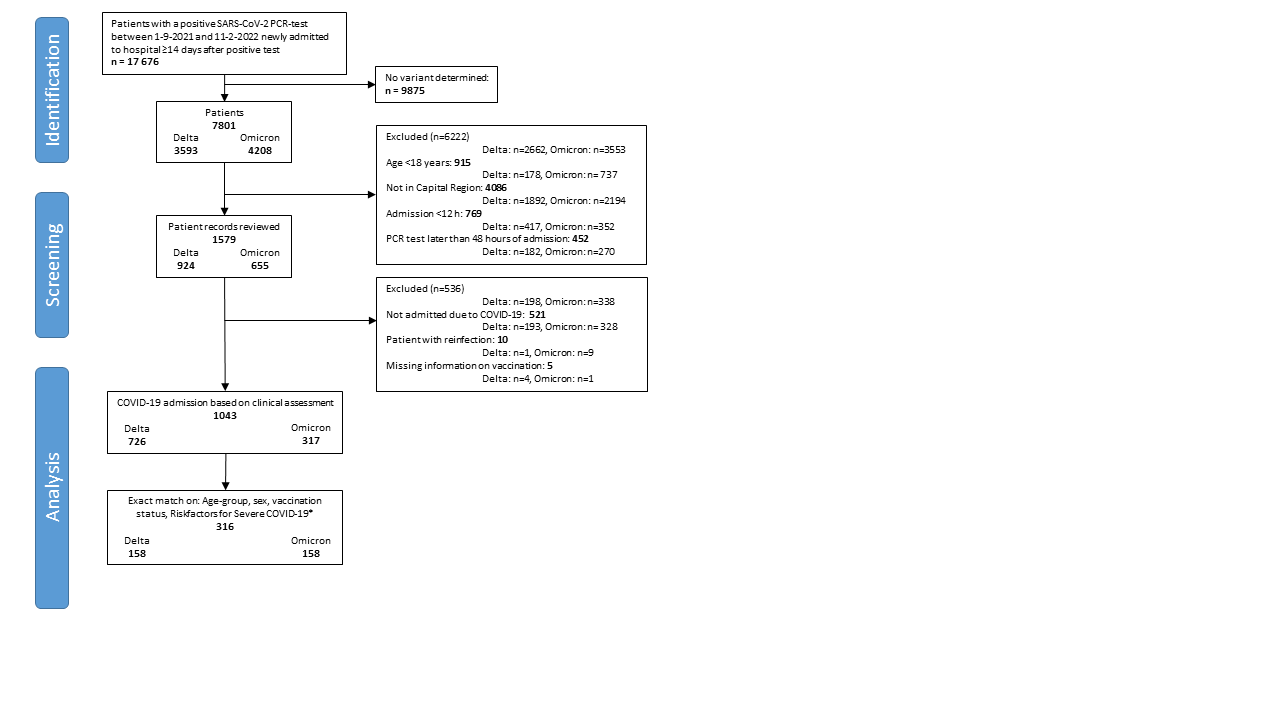


**Supplementary Table A: Baseline characteristics of patients hospitalized with COVID-19 due to SARS-CoV-2 Omicron and Delta variants, according to disease severity. Individuals were matched by age, sex, comorbidities, and vaccination status (Copenhagen, 1 September 2021 to 11 February 2022; *n* = 316)**

|  |  | **Omicron** | | | | **Delta** | | | |  |
| --- | --- | --- | --- | --- | --- | --- | --- | --- | --- | --- |
|  |  | **Mild/Moderate** | **Severe** | **Critical** | **All** | **Mild/Moderate** | **Severe** | **Critical** | **All** | ***p*-value** |
| **Total** |  | 77 (48.7%) | 58 (36.7%) | 23 (14.6%) | 158 (50%) | 56 (35.4%) | 66 (41.8%) | 36 (22.8%) | 158 (50%) |  |
| **Age, median (IQR)** |  | 63 (46 - 77) | 77 (71 - 84) | 72 (58.5 - 77.5) | 72 (58 - 79) | 63 (45.8 - 76.2) | 75 (61.8 - 80.8) | 73 (64.5 - 81) | 73 (56.2 - 79) |  |
| **Sex** | *Male* | 31 (40.3%) | 28 (48.3%) | 15 (65.2%) | 74 (46.8%) | 24 (42.9%) | 31 (47%) | 19 (52.8%) | 74 (46.8%) |  |
|  | *Female* | 46 (59.7%) | 30 (51.7%) | 8 (34.8%) | 84 (53.2%) | 32 (57.1%) | 35 (53%) | 17 (47.2%) | 84 (53.2%) |  |
| **BMI, median (IQR)** |  | 24.4 (21.7 - 28.8) | 23.9 (21.4 - 28) | 24.8 (21.2 - 30) | 24.4 (21.5 - 28.8) | 26.9 (23.2 - 29.6) | 26 (22.9 - 30.9) | 26.3 (21.9 - 30.1) | 26.4 (23 - 30.1) | 0.02 |
| **Immigrated** | *Born abroad* | 19 (24.7%) | 10 (17.2%) | 9 (39.1%) | 38 (24.1%) | 9 (16.1%) | 18 (27.3%) | 12 (33.3%) | 39 (24.7%) | 1 |
|  | *Born in Denmark* | 58 (75.3%) | 48 (82.8%) | 14 (60.9%) | 120 (75.9%) | 47 (83.9%) | 48 (72.7%) | 24 (66.7%) | 119 (75.3%) |  |
| **Smoking** | *Never* | 32 (41.6%) | 14 (24.1%) | 5 (21.7%) | 51 (32.3%) | 18 (32.1%) | 27 (40.9%) | 8 (22.2%) | 53 (33.5%) | 0.60 |
|  | *Current* | 7 (9.1%) | 9 (15.5%) | 3 (13%) | 19 (12%) | 6 (10.7%) | 4 (6.1%) | 4 (11.1%) | 14 (8.9%) |  |
|  | *Previous* | 25 (32.5%) | 27 (46.6%) | 15 (65.2%) | 67 (42.4%) | 17 (30.4%) | 23 (34.8%) | 16 (44.4%) | 56 (35.4%) |  |
|  | *Missing* | 13 (16.9%) | 8 (13.8%) |  | 21 (13.3%) | 15 (26.8%) | 12 (18.2%) | 8 (22.2%) | 35 (22.2%) |  |
| **Frailty** | *No need for daily help* | 48 (62.3%) | 23 (39.7%) | 7 (30.4%) | 78 (49.4%) | 43 (76.8%) | 41 (62.1%) | 22 (61.1%) | 106 (67.1%) | <0.001 |
|  | *Limited need for help* | 20 (26%) | 17 (29.3%) | 7 (30.4%) | 44 (27.8%) | 9 (16.1%) | 17 (25.8%) | 10 (27.8%) | 36 (22.8%) |  |
|  | *Full need for help on a daily basis/lives in a nursing home* | 8 (10.4%) | 18 (31%) | 9 (39.1%) | 35 (22.2%) | 3 (5.4%) | 7 (10.6%) | 3 (8.3%) | 13 (8.2%) |  |
| **COVID-19 vaccination status** | *None* | 24 (31.2%) | 14 (24.1%) | 11 (47.8%) | 49 (31%) | 11 (19.6%) | 24 (36.4%) | 14 (38.9%) | 49 (31%) |  |
|  | *One dose* | 2 (2.6%) |  | 1 (4.3%) | 3 (1.9%) | 2 (3.6%) |  | 1 (2.8%) | 3 (1.9%) |  |
|  | *Two doses* | 26 (33.8%) | 26 (44.8%) | 5 (21.7%) | 57 (36.1%) | 23 (41.1%) | 24 (36.4%) | 10 (27.8%) | 57 (36.1%) |  |
|  | *Three doses* | 25 (32.5%) | 18 (31%) | 6 (26.1%) | 49 (31%) | 20 (35.7%) | 18 (27.3%) | 11 (30.6%) | 49 (31%) |  |
| **Time from vaccination to admission in weeks, median (IQR)** | *weeks* | 19.9 (12.7 – 26.4) | 20.6 (13.5 – 36.6) | 19.6 (15.6 – 38.8) | 19.9 (12.9 – 32) | 17 (8.7 – 27.6) | 21 (10 – 28.5) | 19.4 (10.2 – 29) | 18.3 (9.1 – 28.3) | 0.04 |
| **Charlson comorbidity index – Quan, median (IQR)** |  | 1 (0 – 2.2) | 2 (0.8 – 3) | 2 (1 – 2) | 2 (0 – 3) | 1 (0 – 3) | 1 (0 – 3) | 2 (0 – 2) | 1 (0 – 3) | 0.60 |
| **Charlson comorbidity index – Quan, categorical** | *0* | 29 (37.7%) | 16 (27.6%) | 5 (21.7%) | 50 (31.6%) | 20 (35.7%) | 25 (37.9%) | 10 (27.8%) | 55 (34.8%) | 0.84 |
|  | *1-2* | 29 (37.7%) | 23 (39.7%) | 13 (56.5%) | 65 (41.1%) | 20 (35.7%) | 24 (36.4%) | 18 (50%) | 62 (39.2%) |  |
|  | *3+* | 19 (24.7%) | 19 (32.8%) | 5 (21.7%) | 43 (27.2%) | 16 (28.6%) | 17 (25.8%) | 8 (22.2%) | 41 (25.9%) |  |
| **Severe COVID-19 risk factors** | *Pregnancy* | 7 (9.1%) |  |  | 7 (4.4%) | 3 (5.4%) | 2 (3%) | 1 (2.8%) | 6 (3.8%) | 1 |
|  | *Diabetes mellitus* | 14 (18.2%) | 15 (25.9%) | 9 (39.1%) | 38 (24.1%) | 11 (19.6%) | 20 (30.3%) | 11 (30.6%) | 42 (26.6%) | 0.70 |
|  | *BMI>30* | 10 (13%) | 7 (12.1%) | 7 (30.4%) | 24 (15.2%) | 12 (21.4%) | 15 (22.7%) | 8 (22.2%) | 35 (22.2%) | 0.15 |
|  | *Chronic cardiac disease* | 33 (42.9%) | 36 (62.1%) | 10 (43.5%) | 79 (50%) | 20 (35.7%) | 29 (43.9%) | 17 (47.2%) | 66 (41.8%) | 0.18 |
|  | *Chronic pulmonary disease* | 20 (26%) | 21 (36.2%) | 6 (26.1%) | 47 (29.7%) | 16 (28.6%) | 22 (33.3%) | 11 (30.6%) | 49 (31%) | 0.90 |
|  | *Chronic kidney disease* | 5 (6.5%) | 10 (17.2%) | 3 (13%) | 18 (11.4%) | 7 (12.5%) | 4 (6.1%) | 5 (13.9%) | 16 (10.1%) | 0.86 |
|  | *Chronic liver disease* |  | 1 (1.7%) | 2 (8.7%) | 3 (1.9%) | 3 (5.4%) | 1 (1.5%) |  | 4 (2.5%) | 1 |
|  | *Acute cancer* | 13 (16.9%) | 10 (17.2%) | 6 (26.1%) | 29 (18.4%) | 6 (10.7%) | 7 (10.6%) | 6 (16.7%) | 19 (12%) | 0.86 |
|  | *Immunosuppression* | 12 (15.6%) | 7 (12.1%) | 5 (21.7%) | 24 (15.2%) | 17 (30.4%) | 13 (19.7%) | 6 (16.7%) | 36 (22.8%) | 0.11 |
|  | *Cerebrovascular disease* | 10 (13%) | 18 (31%) | 6 (26.1%) | 34 (21.5%) | 10 (17.9%) | 12 (18.2%) | 2 (5.6%) | 24 (15.2%) | 0.15 |
| **Number of severe COVID-19 risk factors, mean (IQR)** |  | 1.6 (1 – 2) | 2.2 (1 – 3) | 2.3 (1 – 3) | 1.9 (1 – 3) | 1.9 (1 – 3) | 1.9 (1 – 3) | 1.9 (1 – 3) | 1.9 (1 – 3) | 0.08 |
| **Time from symptoms to admission, median (IQR)** |  | 1 (0 – 3) | 3 (0 – 5) | 4 (2 – 7) | 2 (0 – 4.2) | 3 (1 – 9) | 5 (2.8 – 7) | 5 (2.5 – 7) | 5 (2 – 7) | <0.001 |

**Supplementary Table B: Laboratory Results of patients hospitalized with COVID-19 due to SARS-CoV-2 Omicron and Delta variants, according to disease severity. Individuals were matched by age, sex, comorbidities and vaccination status (Copenhagen, 1 September 2021 to 11 February 2022; *n* = 316)**

|  | **Omicron** | | | | **Delta** | | | |  |
| --- | --- | --- | --- | --- | --- | --- | --- | --- | --- |
|  | **Mild/Moderate** | **Severe** | **Critical** | **All** | **Mild/Moderate** | **Severe** | **Critical** | **All** | **p-value** |
| **Total patients** | 77 (48.7%) | 58 (36.7%) | 23 (14.6%) | 158 (100%) | 56 (35.4%) | 66 (41.8%) | 36 (22.8%) | 158 (100%) |  |
|  |  |  |  |  |  |  |  |  |  |
| **Biochemistry** |  |  |  |  |  |  |  |  |  |
| *CRP, median (IQR)** | 30.5 (8 - 68.5) | 99.5 (47 - 153) | 182 (114 - 232.5) | 63 (25 - 132) | 37.5 (19.8 - 90) | 87 (51 - 136.2) | 145 (85.8 - 200.5) | 84 (37.5 - 140) | 0.61 |
| *D-dimer, median (IQR)** | 1 (0.4 - 3) | 2 (1 - 3.4) | 2.3 (1.2 - 5.8) | 1.9 (1 - 3.5) | 0.6 (0.5 - 0.7) | 0.9 (0.6 - 1.6) | 2 (1.3 - 3.1) | 1.1 (0.7 - 2) | 0.11 |
| *Ferritin, median (IQR)** | 160 (47 - 319) | 305 (128 - 717) | 331 (191 - 786) | 256.5 (99 - 592) | 217 (86 - 821) | 434 (206 - 1358) | 1205 (417 - 1618) | 841.5 (184 - 1545) | <0.001 |
| *Procalcitonin, median (IQR)** | 0.1 (0 - 0.2) | 0.1 (0.1 - 1.3) | 0.6 (0.3 - 11.8) | 0.2 (0.1 - 1.1) |  | 0.3 (0.1 - 0.5) | 0.3 (0.2 - 1) | 0.3 (0.1 - 0.8) | 0.85 |
|  |  |  |  |  |  |  |  |  |  |
| **Microbiological findings** |  |  |  |  |  |  |  |  |  |
| *Positive blood cultures* | 1 (1.3%) | 5 (8.6%) | 5 (21.7%) | 11 (7%) |  | 2 (3%) | 10 (27.8%) | 12 (7.6%) | 0.98 |
| *Positive respiratory cultures* | 3 (3.9%) | 11 (19%) | 12 (52.2%) | 26 (16.5%) | 1 (1.8%) | 5 (7.6%) | 7 (19.4%) | 13 (8.2%) | 0.03 |
| *Atypical bacterial pneumonia*** |  | 1 (1.7%) |  | 1 (0.6%) |  |  |  |  | 1 |
|  |  |  |  |  |  |  |  |  |  |
| **Chest X-ray performed in** | 39 (50.6%) | 53 (91.4%) | 21 (91.3%) | 113 (71.5%) | 35 (62.5%) | 58 (87.9%) | 34 (94.4%) | 127 (80.4%) | 0.09 |
| **Chest X-ray findings** |  |  |  |  |  |  |  |  |  |
| *Normal* | 23 (59%) | 12 (22.6%) | 1 (4.8%) | 36 (31.9%) | 18 (51.4%) | 15 (25.9%) |  | 33 (26.0%) | 0.79 |
| *Unilateral /bilateral pulmonary infiltrates* | 10 (26%) | 28 (52.8%) | 19 (86.4%) | 57 (50.4%) | 13 (37.1%) | 42 (72.4%) | 34 (100%) | 89 (70.1%) | <0.001 |
| *Pleural effusion* | 2 (2.6%) | 7 (12.1%) | 5 (21.7%) | 14 (8.9%) | 2 (3.6%) | 3 (4.5%) |  | 5 (3.2%) | 0.06 |
| *Pulmonary edema* | 1 (1.3%) | 3 (5.2%) | 3 (13%) | 7 (4.4%) |  | 1 (1.5%) | 2 (5.6%) | 3 (1.9%) | 0.34 |
| *Other findings* | 6 (7.8%) | 7 (12.1%) |  | 13 (8.2%) | 2 (3.6%) | 3 (4.5%) | 1 (2.8%) | 6 (3.8%) | 0.15 |
|  |  |  |  |  |  |  |  |  |  |
| **Chest CT scan performed in** | 12 (15.6%) | 13 (22.4%) | 8 (34.8%) | 33 (20.9%) | 3 (5.4%) | 8 (12.1%) | 19 (52.8%) | 30 (19%) | 0.01 |
| **Chest CT scan findings** |  |  |  |  |  |  |  |  |  |
| *Normal CT* | 4 (5.2%) | 3 (5.2%) |  | 7 (4.4%) | 1 (1.8%) |  |  | 1 (0.6%) | 0.07 |
| *Unilateral subpleural ground glass opacities* |  |  | 1 (4.3%) | 1 (0.6%) |  | 1 (1.5%) | 1 (2.8%) | 2 (1.3%) | 1 |
| *Bilateral subpleural ground glass opacities with or without consolidation* | 2 (2.6%) | 4 (6.9%) | 6 (26.1%) | 12 (7.6%) | 2 (3.6%) | 8 (12.1%) | 17 (47.2%) | 27 (17.1%) | 0.02 |
| *Unilateral infiltrates with consolidation* | 2 (2.6%) |  | 2 (8.7%) | 4 (2.5%) |  |  |  |  | 0.12 |
| *Pulmonary thromboembolism (central or peripheral)* | 2 (2.6%) | 1 (1.7%) |  | 3 (1.9%) |  |  | 3 (8.3%) | 3 (1.9%) | 1 |
| *Plural effusion* | 2 (2.6%) | 3 (5.2%) | 2 (8.7%) | 7 (4.4%) |  |  | 1 (2.8%) | 1 (0.6%) | 0.07 |
| *Other CT* | 3 (3.9%) | 5 (8.6%) | 3 (13%) | 11 (7%) |  |  | 1 (2.8%) | 1 (0.6%) | 0.01 |

*Peak value during admission. **PCR positive (throat swab or endotracheal aspirate) for *Legionella pneumophila*, *Mycoplasma pneumoniae,* or *Chlamydia pneumoniae.*

**Supplementary Table C: Outcomes in hospital and mortality among patients hospitalized with COVID-19 due to SARS-CoV-2 Omicron and Delta variants, according to disease severity. Individuals were matched by age, sex, comorbidities, and vaccination status (Copenhagen, 1 September 2021 to 11 February 2022 (*n* = 316)**

|  |  | **Omicron** | | | | | **Delta** | | | |  |
| --- | --- | --- | --- | --- | --- | --- | --- | --- | --- | --- | --- |
|  |  | **Mild/Moderate** | **Severe** | **Critical** | **All** | | **Mild/Moderate** | **Severe** | **Critical** | **All** | ***p*-value** |
| **Total** |  | 77 (48.7%) | 58 (36.7%) | 23 (14.6%) | 158 (100%) | | 56 (35.4%) | 66 (41.8%) | 36 (22.8%) | 158 (100%) |  |
| **Oxygen supplementation** | *No oxygen* | 71 (92.2%) | 4 (6.9%) |  | 75 (47.5%) | 54 (96.4%) | | 3 (4.5%) |  | 57 (36.1%) | 0.04 |
|  | *Less than 10 L/min* | 6 (7.8%) | 48 (82.8%) |  | 54 (34.2%) | 2 (3.6%) | | 58 (87.9%) | 1 (2.8%) | 61 (38.6%) |  |
|  | *More than 10 L/min administered by HFNC, NIV, CPAP** |  | 6 (10.3%) | 23 (100%) | 29 (18.4%) |  | | 5 (7.6%) | 35 (97.2%) | 40 (25.3%) |  |
| **Remdesivir** | *Yes* | 19 (24.7%) | 40 (69%) | 15 (65.2%) | 74 (46.8%) | | 9 (16.1%) | 45 (68.2%) | 24 (66.7%) | 78 (49.4%) | 0.74 |
| **Dexamethasone** | *Dexamethasone* | 17 (22.1%) | 51 (87.9%) | 20 (87%) | 88 (55.7%) | | 9 (16.1%) | 56 (84.8%) | 34 (94.4%) | 99 (62.7%) | 0.25 |
| **Antibiotics** | *Antibiotics* | 21 (27.3%) | 40 (69%) | 20 (87%) | 81 (51.3%) | | 26 (46.4%) | 36 (54.5%) | 32 (88.9%) | 94 (59.5%) | 0.14 |
| **IL-6 receptor blocker** | *Yes* |  |  | 5 (21.7%) | 5 (3.2%) | |  |  | 7 (19.4%) | 7 (4.4%) | 0.58 |
| **Anticoagulants** | *None* | 46 (59.7%) | 8 (13.8%) | 2 (8.7%) | 56 (35.4%) | | 39 (69.6%) | 4 (6.1%) | 2 (5.6%) | 45 (28.5%) | 0.003 |
|  | *Prophylactic dosage* | 22 (28.6%) | 38 (65.5%) | 17 (73.9%) | 77 (48.7%) | | 16 (28.6%) | 56 (84.8%) | 28 (77.8%) | 100 (63.3%) |  |
|  | *Therapeutic dosage* | 4 (5.2%) | 4 (6.9%) | 2 (8.7%) | 10 (6.3%) | |  | 3 (4.5%) | 5 (13.9%) | 8 (5.1%) |  |
|  | *other dosage* | 5 (6.5%) | 7 (12.1%) | 2 (8.7%) | 14 (8.9%) | | 1 (1.8%) | 1 (1.5%) |  | 2 (1.3%) |  |
|  | *Missing* |  | 1 (1.7%) |  | 1 (0.6%) | |  | 2 (3%) | 1 (2.8%) | 3 (1.9%) |  |
| **Monoclonal antibodies** | *No* | 62 (80.5%) | 53 (91.4%) | 19 (82.6%) | 134 (84.8%) | | 38 (67.9%) | 54 (81.8%) | 28 (77.8%) | 120 (75.9%) | 0.02 |
|  | *Yes, pre-admission* | 1 (1.3%) |  | 1 (4.3%) | 2 (1.3%) | |  |  |  |  |  |
|  | *Yes, during admission* | 13 (16.9%) | 4 (6.9%) | 3 (13%) | 20 (12.7%) | | 16 (28.6%) | 12 (18.2%) | 8 (22.2%) | 36 (22.8%) |  |
|  | *Missing* | 1 (1.3%) | 1 (1.7%) |  | 2 (1.3%) | | 2 (3.6%) |  |  | 2 (1.3%) |  |
| **MIS-A**** | *No* | 77 (100%) | 55 (94.8%) | 22 (95.7%) | 154 (97.5%) | | 53 (94.6%) | 66 (100%) | 35 (97.2%) | 154 (97.5%) | 1 |
|  | *Missing information* |  | 3 (5.2%) | 1 (4.3%) | 4 (2.5%) | | 3 (5.4%) |  | 1 (2.8%) | 4 (2.5%) |  |
| **Admission to ICU***** | *Yes* |  | 2 (3.4%) | 3 (13%) | 5 (3.2%) | |  |  | 17 (47.2%) | 17 (10.8%) | 0.01 |
|  | *Mechanical ventilation* |  |  | 3 (13%) | 3 (1.9%) | |  |  | 13 (36,1%) | 13 (8.2%) | 0.02 |
|  | *ICU length of stay, days* |  | 1 (0.5 - 1.5) | 22 (11.5 - 23) | 2 (1 - 22) | |  |  | 12.5 (8.8 - 14.8) | 12.5 (8.8 - 14.8) | 0.59 |
|  | *Length of mechanical ventilation, days* |  |  | 12 (6.5 - 14.5) | 12 (6.5 - 14.5) | |  |  | 13 (7 - 24) | 13 (7 - 24) | 0.38 |
| **Length of stay, median (IQR) in days** |  | 2 (1 - 3) | 5 (3 - 6.8) | 8 (4.5 - 13) | 3 (1 - 7) | | 2 (1 - 3) | 4 (3 - 6.8) | 14.5 (6.8 - 27.2) | 4 (2 - 8.8) | 0.06 |
| **Readmission within 30 days** | *Yes* | 8 (10.4%) | 7 (12.1%) | 5 (21.7%) | 20 (12.7%) | | 9 (16.1%) | 11 (16.7%) | 2 (5.6%) | 22 (13.9%) | 0.74 |
| **Death within 30 days of admission** |  | 1 (1.3%) | 12 (20.7%) | 12 (52.2%) | 25 (15.8%) | | 3 (5.4%) | 11 (16.7%) | 15 (41.7%) | 29 (18.4%) | 0.65 |
| **Death within 60 days of admission** |  | 3 (3.9%) | 14 (24.1%) | 12 (52.2%) | 29 (18.4%) | | 3 (5.4%) | 12 (18.2%) | 18 (50%) | 33 (20.9%) | 0.67 |

* high-flow nasal cannula, non-invasive ventilation or continuous positive airway pressure, ** multisystem inflammatory syndrome in adults, ***intensive care unit

**Appendix 1.**

*Selection bias:* Delta and Omicron patients included in the source population were drawn from the same administrative databases. To address potential bias associated with geographic location, we limited the source population to patients who had permanent residency within the Capital Region of Denmark and were there for the entire duration of their hospitalization. Thus, we excluded patients transferred from another area because they required a higher level of care. In addition, selection bias may have occurred because only a subgroup of RT-PCR positive samples was sent for sequencing or variant determination. To address any selection bias, when assessing the exposure, we matched Omicron and Delta patients using key variables that may influence outcomes (i.e., age, sex, comorbidities, and number of vaccine doses administered).

*Measurement bias:* we assessed the presence or absence of prognostic factors and outcomes by retrieving information from two independent data sources. Medical doctors were trained in using the standardized data collection instrument. To reduce the information bias related to the collection of data by multiple investigators, medical records were reviewed by two independent physicians. In cases of disagreement, a third physician was consulted. For ICU admissions and death, information was confirmed by comparison with data in the national databases. For the composite outcome, information obtained from medical charts was considered sufficient.

Investigators were initially blinded to exposure data at the verification stage, but information became available to investigators during the assessment of medical records.

**Appendix 2.**

There were no missing data for admission dates, discharge dates, oxygen use, or vital status. We supplemented missing information for CCI with information on comorbidities retrieved from patients’ records; if any risk factors for severe COVID-19 were registered, a CCI of 1–2 was imputed, whereas a CCI of 0 was imputed if there were no risk factors for severe COVID-19. Missing data for matching variables led to exclusion from the matching population.
